# Assays of D-Amino Acid Oxidase Activity

**DOI:** 10.3389/fmolb.2017.00102

**Published:** 2018-01-18

**Authors:** Elena Rosini, Laura Caldinelli, Luciano Piubelli

**Affiliations:** ^1^Department of Biotechnology and Life Sciences, University of Insubria, Varese, Italy; ^2^The Protein Factory Research Center, Politecnico of Milan and University of Insubria, Milan, Italy

**Keywords:** D-amino acids, D-amino acid oxidase, flavoproteins, enzymatic activity, analytical detection

## Abstract

D-amino acid oxidase (DAAO) is a well-known flavoenzyme that catalyzes the oxidative FAD-dependent deamination of D-amino acids. As a result of the absolute stereoselectivity and broad substrate specificity, microbial DAAOs have been employed as industrial biocatalysts in the production of semi-synthetic cephalosporins and enantiomerically pure amino acids. Moreover, in mammals, DAAO is present in specific brain areas and degrades D-serine, an endogenous coagonist of the N-methyl-D-aspartate receptors (NMDARs). Dysregulation of D-serine metabolism due to an altered DAAO functionality is related to pathological NMDARs dysfunctions such as in amyotrophic lateral sclerosis and schizophrenia. In this protocol paper, we describe a variety of direct assays based on the determination of molecular oxygen consumption, reduction of alternative electron acceptors, or α-keto acid production, of coupled assays to detect the hydrogen peroxide or the ammonium production, and an indirect assay of the α-keto acid production based on a chemical derivatization. These analytical assays allow the determination of DAAO activity both on recombinant enzyme preparations, in cells, and in tissue samples.

## Introduction

**Figure 1:**
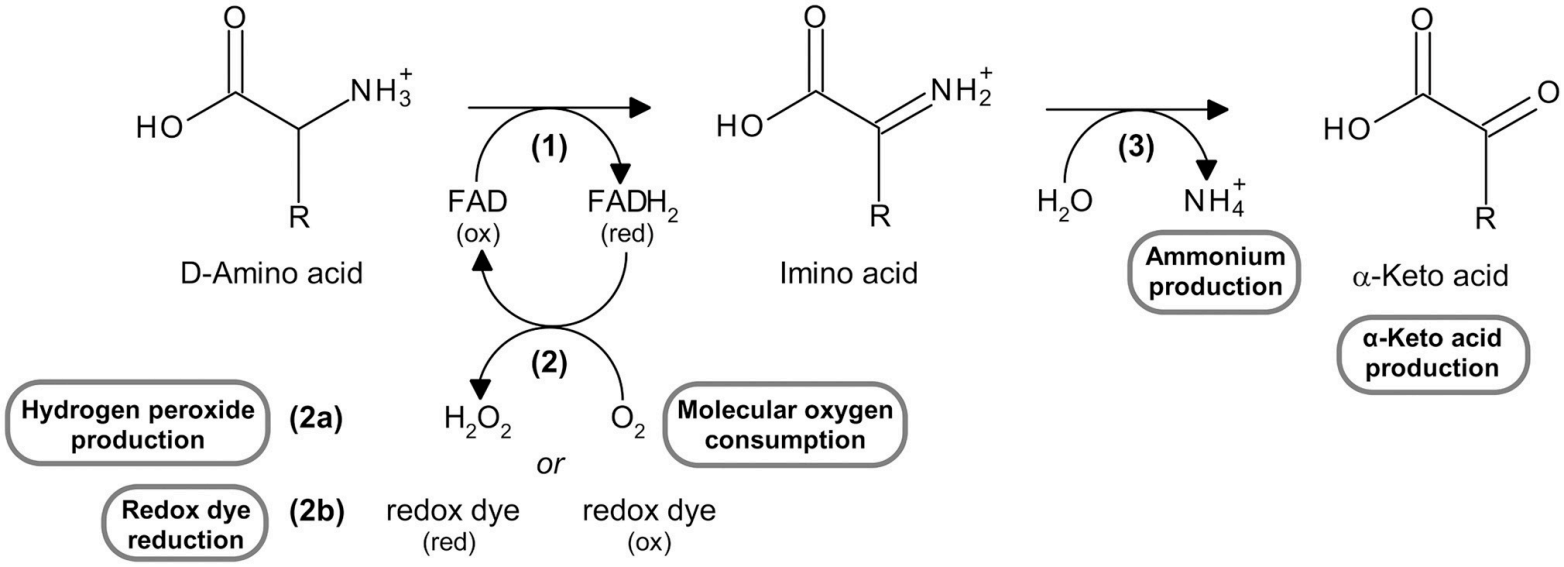
Reaction catalyzed by DAAO, highlighting the substrates and the products which can be measured using the assays reported here. See text for the explanation of the steps in the reaction.

D-Amino acid oxidase (DAAO, EC 1.4.3.3) is a flavoenzyme containing a non-covalently bound FAD molecule per 40 kDa monomer that belongs to the dehydrogenase/oxidase class of flavoproteins (Pollegioni et al., 2007a). DAAO catalyzes the oxidative deamination of the D-isomer of α-amino acids to the corresponding α-keto acids, according to the scheme reported in Figure 1. It shows a strict selectivity toward D-isomers (i.e., it does not oxidize L-amino acids) and possesses a broad substrate specificity: it oxidizes aliphatic, aromatic and polar α-D-amino acids, whereas D-aspartate and D-glutamate are not substrates for DAAO (Pollegioni et al., 2008).

The reaction catalyzed by DAAO can be divided into (Pollegioni et al., 2008):

1. a reductive half-reaction, in which the enzyme catalyzes the dehydrogenation of α-D-amino acids to their imino acid counterparts with concomitant reduction of FAD to FADH_2_ [reaction (1) in Figure 1];
2. an oxidative half-reaction, in which FADH_2_ is reoxidized by molecular oxygen (the physiological electron acceptor) to produce hydrogen peroxide [reaction (2a) in Figure 1]; FADH_2_ can also be reoxidized *in vitro* by artificial electron acceptors, such as some redox dyes [reaction (2b) in Figure 1].
3. The spontaneous hydrolysis of the imino acid to give the corresponding α-keto acid and ammonium [reaction (3) in Figure 1].

DAAO is present in all eukaryotic taxa with the exception of plants (Pollegioni et al., 2007a). All known DAAOs share the same catalytic mechanism, but show important differences in biochemical and structural properties, such as catalytic efficiency, substrate specificity, oligomeric state, stability, kinetic mechanism, and FAD binding. The diverse properties of DAAOs from different sources reflect their physiological role (for an exhaustive review, see Pollegioni et al., 2007a). DAAO is an efficient catabolic enzyme in yeasts where it oxidizes α-D-amino acids, making it possible to use them as carbon, nitrogen, or energy sources. The main role of DAAO in mammals (including humans) is related to its presence in selected brain areas where it is devoted to the catabolism of D-serine. This α-D-amino acid is a neuromodulator acting as a coagonist of the N-methyl-D-aspartate receptors (NMDARs). Dysfunction of this receptor has been correlated to different neurological or psychiatric diseases: an overstimulation of these receptors is involved in stroke, epilepsy, in neurodegenerative pathologies such as Parkinson's and Alzheimer's diseases and amyotrophic lateral sclerosis. An hypostimulation of NMDARs, in contrast, is involved in psychiatric diseases such as schizophrenia, attention-deficit hyperactivity disorder, and chronic depression (Ross et al., 2006; Wu et al., 2007; Mitchell et al., 2010; Pollegioni and Sacchi, 2010; Nagasawa et al., 2012; Sacchi et al., 2012; Zhou and Sheng, 2013). Thus, by modulating D-serine levels, DAAO plays a key role in regulating NMDARs activation state in mammals. Owing to of its role in human brain, molecules that modulate or inhibit DAAO can act as innovative drugs for the treatment of the many diseases linked to NMDARs dysfunction (Sacchi et al., 2013).

Microbial DAAOs possess properties that render them suitable for industrial biotechnological applications: for example, they are stable enzymes and show broad substrate specificity, high turnover number (Table 1), and a tight binding with the FAD cofactor. The production of DAAOs in large amounts as recombinant proteins together with the availability of the 3D-structure (e.g., Mattevi et al., 1996; Mizutani et al., 1996; Umhau et al., 2000; Kawazoe et al., 2006), allowed the design and production by protein engineering techniques of enzyme variants with new and evolved properties (Pollegioni et al., 2007b; Rosini et al., 2008, 2009, 2010; Wang et al., 2008; Wong et al., 2010; Pollegioni and Molla, 2011; Golubev et al., 2014).

**Table 1:** Comparison of kinetic properties of DAAO from different organisms.

	**Yeast DAAOs**		**Mammalian DAAOs**		
	**RgDAAO^a^**	**TvDAAO^a^**	**pkDAAO^a^**	**rDAAO^b^**	**hDAAO^c^**
**Kinetic parameters on D-alanine**^d^					
*k*_*cat*_, _*app*_ (s^−1^)	81 ± 5	46 ± 3	7.3 ± 0.6	27 ± 1	5.2 ± 0.1
*K*_*m*_, _*app*_ (mM)	1.0 ± 0.2	7.0 ± 0.9	1.7 ± 0.3	140 ± 20	1.3 ± 0.2

*RgDAAO, DAAO from Rhodotorula gracilis; TvDAAO, DAAO from Trigonopsis variabilis; pkDAAO, DAAO from pig kidney; rDAAO, DAAO from rat; hDAAO, DAAO from Homo sapiens*.

a *(Pollegioni et al., 2004)*.

b *(Frattini et al., 2011)*.

c *(Molla et al., 2006)*.

d *The activity was determined using the oxygen-consumption assay, at 25°C, pH 8.5, and at air saturation ([O_2_] = 0.253 mM)*.

The main biotechnological applications of DAAO have been reviewed in detail in Pollegioni et al. (2007b, 2008) and Pollegioni and Molla (2011) and comprise: (i) production of 7-amino cephalosporanic acid from cephalosporin C, the starting molecule for the production of semi-synthetic cephalosporins; (ii) resolution of racemic mixtures of amino acids to produce enantiomerically pure natural or synthetic amino acids as fine chemicals; (iii) detection and quantification of D-amino acids in biological samples and foodstuff—D-amino acids are components of the bacterial cell wall and thus their presence in food could indicate bacterial contamination and can be used as an indicator of foodstuff aging; and (iv) use as selective marker in plants.

DAAO activity can be determined using different assays based on: (i) molecular oxygen consumption, directly measured by a Clark-type oxygen electrode (Sacchi et al., 2004; Molla et al., 2006); (ii) reduction of redox dyes used as alternative electron acceptors (instead of molecular oxygen), followed by a colorimetric assay (Brugger et al., 2014); (iii) hydrogen peroxide production, indirectly detected by enzyme-coupled assays in which a chromogenic (*o*-dianisidine or 4-aminoantipyrine; Job et al., 2002; Sacchi et al., 2004; Rosini et al., 2008) or a fluorogenic (Amplex® UltraRed, Invitrogen; Sacchi et al., 2011; Hopkins et al., 2013) substrate is used in combination with horseradish peroxidase, or in which a chromogenic reagent (Purpald®: 4-amino-3-hydrazino-5-mercapto-1,2,4-triazole, Sigma-Aldrich) is employed in combination with catalase (Dickinson and Jacobsen, 1970; Watanabe et al., 1978; Sasabe et al., 2014); (iv) α-keto acid formation, directly detected by measuring the increase in absorbance in the ultraviolet range (Tedeschi et al., 2012) or indirectly detected following the reaction of the α-keto acid produced with 2,4-dinitrophenylhydrazine, to give a chromogenic 2,4-dinitrophenylhydrazone derivative (Nagata et al., 1988); and (v) ammonium production, indirectly detected using an enzyme-coupled assay in which α-ketoglutarate is converted to L-glutamate by the NAD^+^-dependent L-glutamate dehydrogenase (Holme and Goldberg, 1975).

Here, we describe in detail the analytical assays listed above; full lists of materials and equipment required and step-by-step procedures are given, together with examples of application and anticipated results.

## Materials and equipment

Unless otherwise stated, all chemicals and reagents were of analytical grade and were purchased from Sigma-Aldrich (Milan, Italy). Solutions were prepared at room temperature using ultrapure water (e.g., Milli-Q grade water) and assays were carried out at 25°C.

One DAAO unit is defined as the amount of enzyme that converts 1 μmol of D-amino acid per minute at 25°C (Molla et al., 1998). O_2_ concentration at 25°C and at air saturation is 0.253 mM. Unless otherwise stated, in order to obtain reliable specific activity values on different D-amino acids, a substrate concentration 10-fold higher than the *K*_m_ value is used.

### Enzymes

Recombinant RgDAAO and hDAAO were expressed and purified from *Escherichia coli* cells as previously described (Fantinato et al., 2001; Molla et al., 2006). The concentration of purified enzyme preparations was determined by using the extinction coefficient at ~450 nm (12.6 and 12.2 mM^−1^cm^−1^ for RgDAAO and hDAAO, respectively).

### Determination of molecular oxygen consumption: polarographic assay

DAAO activity can be assayed polarographically using an oxygen electrode (Sacchi et al., 2004; Molla et al., 2006).

1. Oxygraph system (Hansatech Instr. Ltd, Pentney, Norfolk, UK): a highly sensitive S1 Clark-type polarographic oxygen electrode disc is mounted in a DW1/AD electrode chamber and connected to the Oxygraph electrode control unit. The electrode disc comprises a central platinum cathode and a concentric circular silver anode. By using the electrode chamber, dissolved oxygen can be measured in liquid-phase samples in the 0.2–2.5 mL volume range. Precise temperature control of the sample and electrode disc (the oxygen solubility decreases as temperature increases) is achieved by connecting the water jacket of the electrode chamber to a thermoregulated circulating water bath. The sample is continuously stirred in order to ensure that the dissolved oxygen is kept evenly distributed throughout the reaction vessel.
2. Electrolyte: 50% saturated solution of potassium chloride.
3. Cigarette Rizla+ Blue regular rolling paper (Rizla).
4. Thin layer of polytetrafluoroethylene (PTFE) membrane, selectively permeable to molecular oxygen (Hansatech Instruments Ltd).
5. Sodium dithionite crystals.
6. Substrates: D-amino acid solutions (at different concentrations, depending on the corresponding *K*_m_ values) in 75 mM (final concentration) disodium pyrophosphate buffer, pH 8.5.

### Determination of the redox dye reduction

Different redox dyes are known for their ability to directly react with the reduced form of the cofactor of flavin-dependent oxidases and dehydrogenases and thus can be used as artificial electron acceptors for the assay of these enzymes. 2,6-Dichlorophenol-indophenol (DCPIP), methylene green (MG), and thionine (Thi) show absorption changes in the visible range between their oxidized and reduced forms and were thus selected to directly detect different enzymatic activities, including DAAO (Brugger et al., 2014).

1. Microtiter plate reader (Infinite 200, Tecan, Cernusco sul Naviglio, Milan, Italy).
2. 96-well microplates.
3. 50 mM phosphate buffer, pH 8.0.
4. Redox dye (electron acceptor) solutions: 3 mM DCPIP or 0.5 mM MG or 1 mM Thi, dissolved in water.
5. Substrates: D-amino acid solutions (at different concentrations, depending on the corresponding *K*_m_ values) in 50 mM phosphate buffer, pH 8.0.

### Determination of hydrogen peroxide production

**Figure 2:**
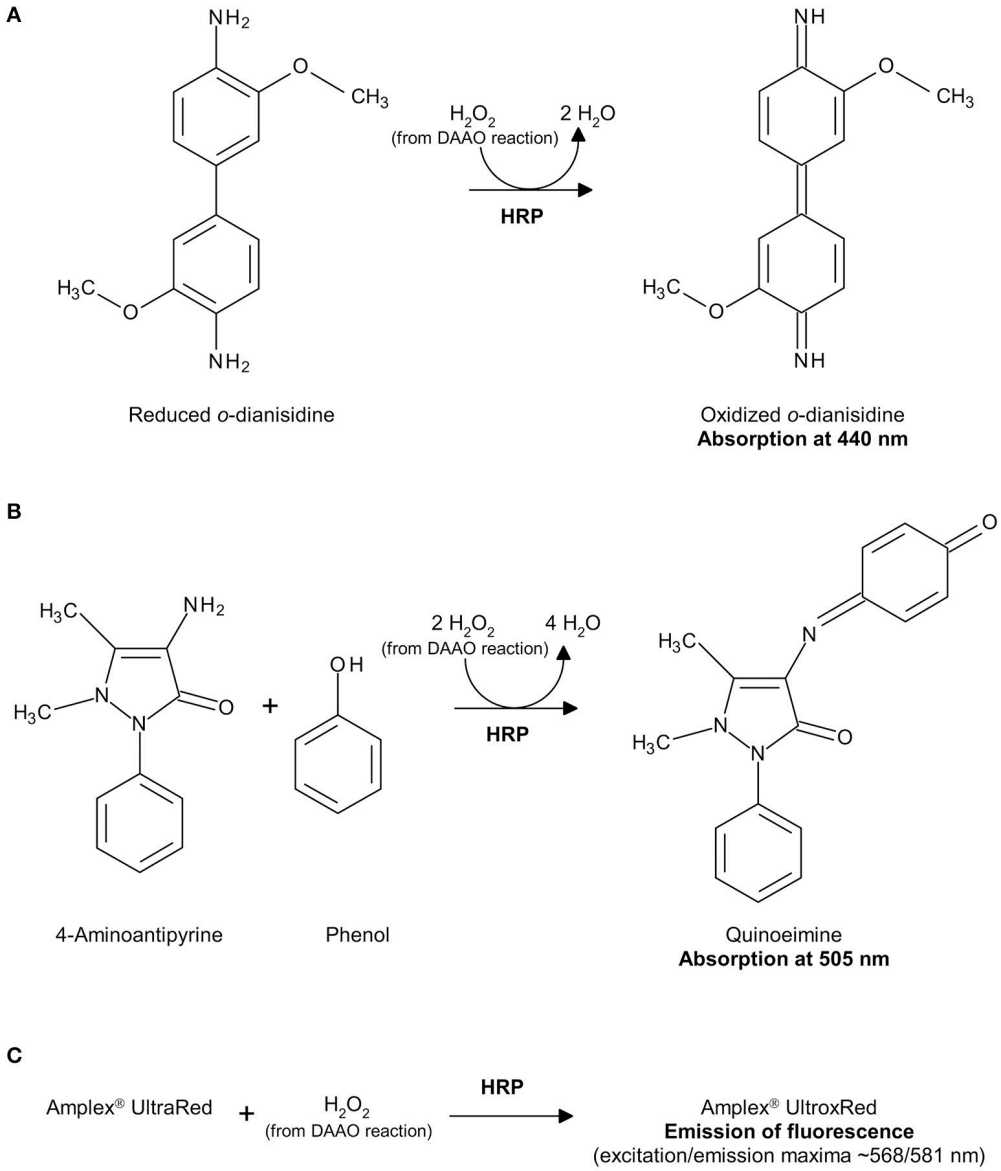
Detection of hydrogen peroxide produced by DAAO in a HRP-coupled reaction using *o*-DNS **(A)**, 4-AAP **(B)** or Amplex® UltraRed Reagent **(C)**.

**Figure 3:**
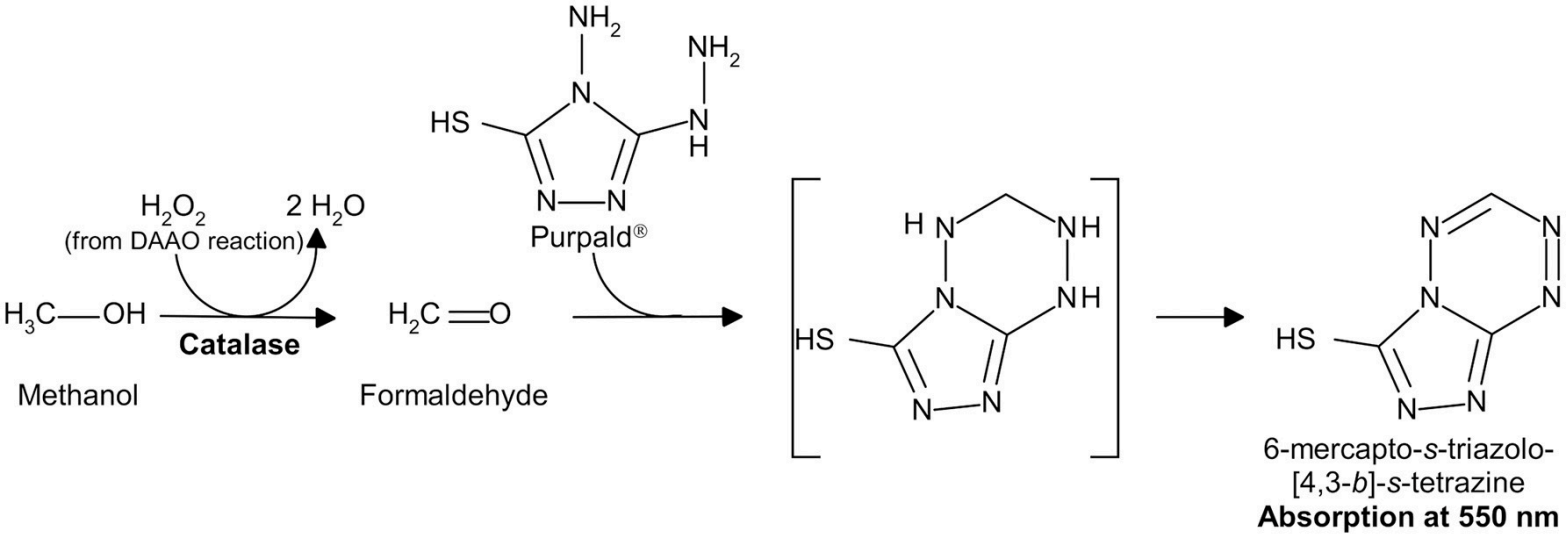
Detection of hydrogen peroxide produced by DAAO in a catalase-coupled reaction using Purpald®.

Hydrogen peroxide can be detected by a second enzyme-coupled reaction in which the chromogenic *o*-dianisidine (*o*-DNS) (Sacchi et al., 2004; Rosini et al., 2008) or 4-aminoantipyrine (4-AAP) (Job et al., 2002) or the fluorogenic Amplex® UltraRed Reagent (Invitrogen, Thermo-Fisher Sci. Co., Carlsbad, CA, USA; Sacchi et al., 2011; Hopkins et al., 2013) reagent substrates are used together with horseradish peroxidase (HRP, EC 1.11.1.7; Roche, Mannheim, Germany; specific activity: ~225 U/mg lyophilized. One unit of HRP is defined as the amount of enzyme that converts 1 μmol of hydrogen peroxide per minute at 25°C in the presence of guaiacol; Figure 2), or the chromogenic Purpald® reagent is used together with catalase (EC 1.11.1.6; Genencor, Palo Alto, CA, USA; one unit of catalase is defined as the amount of enzyme which produces 1 μmol of hydrogen peroxide per minute at 30°C, pH 6.8; Figure 3; Dickinson and Jacobsen, 1970; Watanabe et al., 1978; Sasabe et al., 2014).

**(A) Horseradish Peroxidase and** ***o*-Dianisidine Coupled Assay**

1. UV/Vis spectrophotometer (V-560, Jasco, Cremella, Lecco, Italy) connected to a water bath for temperature control or a microtiter plate reader (Infinite 200, Tecan).
2. 1.5 mL plastic disposable microcuvettes or 96-well microplates.
3. Automated liquid handling system (epMotion® 5075, Eppendorf, Milan, Italy).
4. 100 mM disodium pyrophosphate buffer, pH 8.5.
5. 10 mM *o*-DNS dihydrochloride in water. The solution must be freshly prepared every day and kept on ice.
6. 100 U/mL HRP in 100 mM disodium pyrophosphate buffer, pH 8.5. A concentrated stock solution (e.g., 2,500 U/mL) can be stored at 4°C for several months; the diluted working solution must be freshly prepared every day and kept on ice.
7. Substrates: D-amino acid solutions (at different concentrations, depending on the corresponding *K*_m_ values) in 100 mM disodium pyrophosphate buffer, pH 8.5.
8. Lysis buffer: 50 mM disodium pyrophosphate buffer, pH 8.5, 1 mM EDTA, 100 mM NaCl, 40 μg/mL lysozyme (from chicken egg white), and 1 μg/mL DNaseI (from bovine pancreas; Roche), all final concentrations. Lysozyme and DNaseI must be freshly added from appropriate stock solutions.

**(B) Horseradish Peroxidase and 4-Aminoantipyrine Coupled Assay**

1. UV/Vis spectrophotometer (V-560, Jasco) connected to a water bath for temperature control or a microtiter plate reader (Infinite 200, Tecan).
2. 1.5 mL plastic disposable microcuvettes or 96-well microplates.
3. Automated liquid handling system (epMotion® 5075, Eppendorf).
4. 100 mM disodium pyrophosphate buffer, pH 8.5.
5. 15 mM 4-AAP solution in water. The solution must be freshly prepared every day and kept on ice.
6. 200 mM phenol.
7. 250 U/mL HRP in 100 mM disodium pyrophosphate buffer, pH 8.5. A concentrated stock solution (e.g., 2,500 U/mL) can be stored at 4°C for several months; the diluted working solution must be freshly prepared every day and kept on ice.
8. Substrates: D-amino acid solutions (at different concentrations, depending on the corresponding *K*_m_ values) in 100 mM disodium pyrophosphate buffer, pH 8.5.
9. Lysis buffer: 50 mM disodium pyrophosphate buffer, pH 8.5, 1 mM EDTA, 100 mM NaCl, 40 μg/mL lysozyme, and 1 μg/mL DNaseI (all final concentrations). Lysozyme and DNaseI must be freshly added from appropriate stock solutions.

**(C) Horseradish Peroxidase and Amplex**^®^ **Ultrared Coupled Assay**

1. Microtiter plate reader (Infinite 200, Tecan).
2. Black 96-well microplates.
3. 50 mM sodium phosphate buffer, pH 7.4.
4. 10 U/mL HRP in 50 mM sodium phosphate buffer, pH 7.4. A concentrated stock solution (e.g., 2,500 U/mL) can be stored at 4°C for several months; the diluted working solution must be freshly prepared every day and kept on ice.
5. Substrates: D-amino acid solutions (at different concentrations, depending on the corresponding *K*_m_ values) in 50 mM sodium phosphate buffer, pH 7.4.
6. Inhibitor stock solutions: different compounds dissolved in dimethyl sulfoxide at 10 mM final concentration and stored at 4°C.
7. Lysis buffer: 50 mM sodium phosphate buffer, pH 7.4, 1 μM pepstatin, 2 μM leupeptin, 500 μM phenylmethylsulfonyl fluoride (PMSF), 10 μM FAD (all final concentrations). Pepstatin, leupeptin, PMSF, and FAD must be freshly added from appropriate stock solutions.
8. 0.5 M sodium azide in 50 mM sodium phosphate buffer, pH 7.4. This solution can be stored at room temperature for several months.
9. 500 mM sodium benzoate in 50 mM sodium phosphate buffer, pH 7.4.
10. 1 mM and 10 mM FAD solutions in 50 mM sodium phosphate buffer, pH 7.4.
11. Amplex® UltraRed Reagent solution: allow this reagent to equilibrate at room temperature before use. Prepare a 10 mM stock solution adding 340 μL of dimethyl sulfoxide to the content of one vial (each vial contains 1 mg of the lyophilized powder) and vortex. Prepare 25 μL aliquots and store in the dark at −20°C.
12. Amplex® UltraRed Stop Reagent solution: dissolve the content of the Stop Reagent vial in 1.45 mL of ethanol. Vortex briefly and mix 1 mL of this solution with 1 mL of water. The solution must be freshly prepared every day and stored in the dark at 4°C. The solution is colorless; the appearance of dark coloration is indicative of decomposition.
13. Working solutions (WS): a different working solution is required for each application:
  ***(a) Detection of DAAO activity of purified protein preparations on*** ***different D-amino acids***
    **WS**_**A**_**:** mix 50 μL of 10 mM Amplex® UltraRed Reagent solution and 100 μL of 10 U/mL HRP in 5 mL (final volume) of 50 mM sodium phosphate buffer, pH 7.4;
  ***(b) Detection of DAAO activity in U87 cells*.** For this application, three different WS are required:
    **WS**_**B1**_**:** add 150 μL of 400 mM D-serine, 75 μL of 1 mM FAD and 30 μL of 500 mM sodium azide to 1.2 mL of 50 mM sodium phosphate buffer, pH 7.4. Just before aliquoting the working solution in the wells, add 15 μL of 10 mM Amplex® UltraRed Reagent solution and 30 μL of 10 U/mL HRP. The final volume is 1.5 mL;
    **WS**_**B2**_**:** to be used for the negative control without substrate. This solution has the same composition as WS_B1_, except that D-serine is replaced by 150 μL of buffer (50 mM sodium phosphate buffer, pH 7.4);
    **WS**_**B3**_**:** to be used for the negative control in the presence of benzoate (a well-known DAAO inhibitor): 9 μL of 500 mM sodium benzoate are added to 1.5 mL of WS_B1_ (final sodium benzoate concentration: 3 mM).
  ***(c) Identification of hDAAO Inhibitors***
    **WS**_**C**_**:** mix 220 μL of 1 M D-serine, 4 μL of 10 mM FAD, 100 μL of 10 mM Amplex® UltraRed Reagent solution, and 200 μL of 10 U/mL HRP in 10 mL (final volume) of 50 mM sodium phosphate buffer, pH 7.4.
14. Hydrogen peroxide stock solution 30% (w/w) in water (9.8 M): the diluted solutions (10–100 μM, to be used for the calibration curves) must be freshly prepared every day.

**(D) Catalase and Purpald**^®^ **Coupled Assay**

1. UV/Vis spectrophotometer (V-560, Jasco) connected to a water bath for temperature control.
2. 1.5 mL plastic disposable microcuvettes.
3. 5 mL plastic tubes.
4. 133 mM sodium pyrophosphate buffer, pH 8.3.
5. 34 mM Purpald® in 0.5 M HCl.
6. 0.1 mM FAD in 133 mM sodium pyrophosphate buffer, pH 8.3.
7. 0.75% (w/v) KIO_4_ in 0.2 M KOH.
8. 70% (v/v) methanol.
9. 10% (w/v) trichloroacetic acid.
10. 5 M KOH.
11. 700 U/mL catalase in 133 mM sodium pyrophosphate buffer, pH 8.3. The solution must be freshly prepared every day and kept on ice.
12. Substrates: D-amino acid solutions (at different concentrations, depending on the corresponding *K*_m_ values) in 133 mM sodium pyrophosphate buffer, pH 8.3.
13. Human post mortem tissue samples from different regions of the central nervous system (CNS).

### Determination of α-Keto acid production

**Figure 4:**
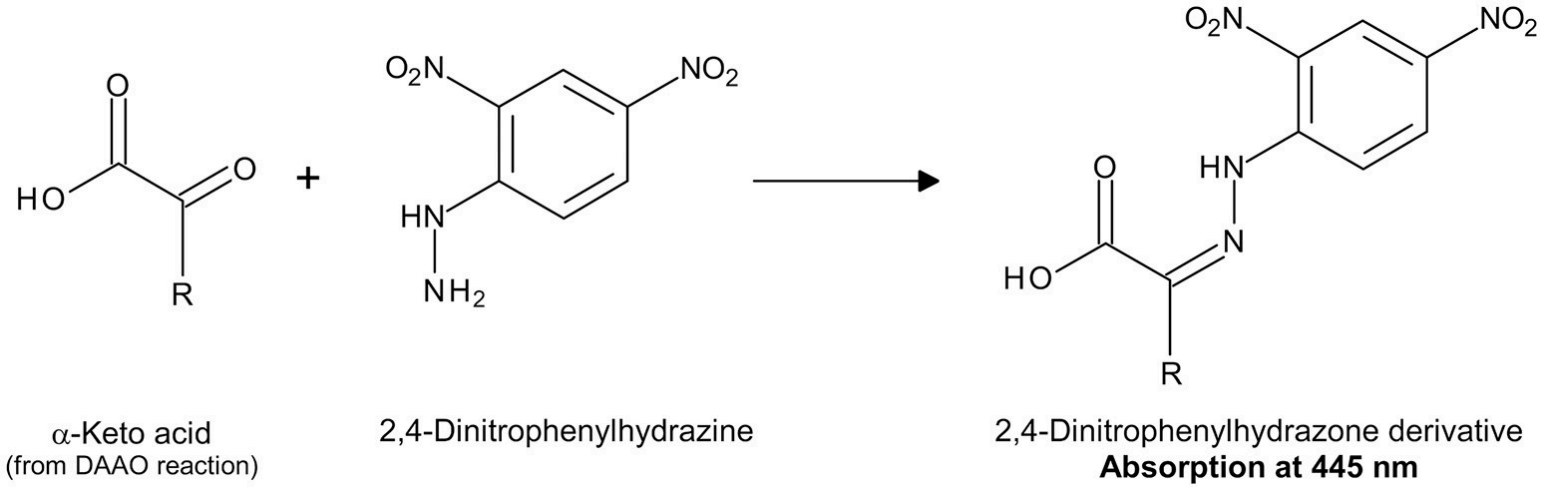
Detection of α-keto acids by derivatization with DNP.

α-Keto acids concentration can be determined by measuring their absorbance in the ultraviolet range (Tedeschi et al., 2012) or following their chemical derivatization with 2,4-dinitrophenylhydrazine (DNP) to produce the corresponding 2,4-dinitrophenylhydrazone derivatives, which show an absorption maximum at 445 nm (Figure 4; Nagata et al., 1988).

**A) Direct Spectrophotometric Assay**

1. UV/Vis spectrophotometer (V-560, Jasco) connected to a water bath for temperature control.
2. 1.5 mL quartz microcuvettes.
3. 75 mM disodium pyrophosphate buffer, pH 8.5.
4. α-Keto acid solution in 75 mM disodium pyrophosphate buffer, pH 8.5 (for the calibration curve).
5. Substrates: D-amino acid solutions (at different concentrations, depending on the corresponding *K*_m_ values) in 75 mM disodium pyrophosphate buffer, pH 8.5.

**B) Indirect Assay with 2,4-Dinitrophenylhydrazine**

1. UV/Vis spectrophotometer (V-560, Jasco) connected to a water bath for temperature control.
2. 1.5 mL glass or plastic disposable microcuvettes.
3. 1 mM DNP solution in 1 M HCl.
4. 0.6 M NaOH.
5. 75 mM disodium pyrophosphate buffer, pH 8.5.
6. α-Keto acid solution in 75 mM disodium pyrophosphate buffer, pH 8.5 (for the calibration curve).
7. Substrates: D-amino acid solutions (at different concentrations, depending on the corresponding *K*_m_ values) in 75 mM disodium pyrophosphate buffer, pH 8.5.

### Determination of ammonium production: glutamate dehydrogenase and α-ketoglutarate coupled assay

**Figure 5:**
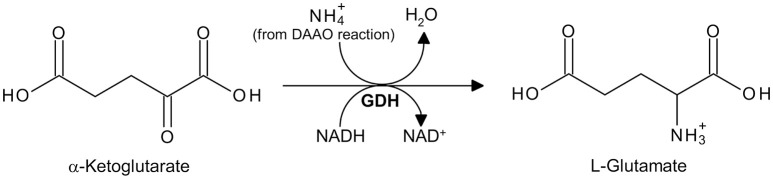
Detection of ammonium produced by DAAO in a GDH-coupled reaction using α-ketoglutarate.

The ammonium produced by DAAO reaction on a D-amino acid can be detected by a second enzyme-coupled reaction in which α-ketoglutarate is converted to L-glutamate by L-glutamate dehydrogenase from bovine liver (GDH, EC 1.4.1.3; specific activity ≥35 U/mg protein. One GDH unit is defined as the amount of enzyme that reduces 1 μmol of α-ketoglutarate to L-glutamate per minute at pH 7.3 and 25°C, in the presence of ammonium ions) and NADH is oxidized to NAD^+^ (Figure 5; Holme and Goldberg, 1975). The decrease of absorbance at 340 nm, due to the conversion of NADH to NAD^+^, is followed.

1. UV/Vis spectrophotometer (V-560, Jasco) connected to a water bath for temperature control.
2. 1.5 mL quartz microcuvettes.
3. 1,000 U/mL GDH in 75 mM disodium pyrophosphate buffer, pH 8.5.
4. 75 mM disodium pyrophosphate buffer, pH 8.5.
5. 5 mM α-ketoglutarate in 75 mM disodium pyrophosphate buffer, pH 8.5.
6. 0.25 mM NADH.
7. Substrates: D-amino acid solutions (at different concentrations, depending on the corresponding *K*_m_ values) in 75 mM disodium pyrophosphate buffer, pH 8.5.

## Stepwise procedures

### Determination of molecular oxygen consumption: polarographic assay

1. Preparation of the electrode disc: before use, a small drop of electrolyte is placed on top of the dome of the central platinum cathode of the electrode disc and a 1.5 cm^2^ cigarette Rizla+ Blue rolling paper spacer is placed over the electrolyte. The paper spacer is covered with a similar sized piece of thin, oxygen-permeable polytetrafluoroethylene membrane. Once prepared, the disc is mounted in the base of the electrode chamber and 1 mL of deionized water is added in the chamber.
2. Calibration: the disc is connected to the electrode control unit, which applies a polarizing voltage (0.7 V) between the platinum and silver electrodes and generates a small current proportional to oxygen concentration in the sample. The amperometric value measured with 1 mL of stirred air-saturated water is considered as 100% of oxygen content, and the amperometric value measured after adding a few crystals of sodium dithionite corresponds to 0% of oxygen content.
3. Measurement of DAAO activity: 1 mL of the substrate solution is placed in the electrode chamber, thermostated at 25°C, and the registration of amperometric signal is started. Once the recorded signal is stable, 10 μL of DAAO solution (~0.05 U) is added. The DAAO activity is calculated from the initial (1–2 min) oxygen consumption rate (μmol/min) using the following equation:
  $$\begin{array}{l} \frac{\text{U}}{\text{m}{\text{L}}_{\text{DAAO}}}=\frac{{\text{μmol}}_{{\text{O}}_{2}}/\text{min}}{\text{m}{\text{L}}_{\text{DAAO}}}\;\times \text{m}{\text{L}}_{\text{TOT}} \end{array}$$
  where mL_TOT_ is the final volume of the reaction mixture (1.01 mL). To accurately determine the DAAO activity, the initial oxygen consumption rate should be in the 0.02–0.05 μmol/min range. The detection range is reported in Table **3**.

#### Application: determination of the steady-state kinetic parameters and substrate specificity

The apparent kinetic parameters are investigated by this assay, employing different concentrations of each D-amino acid as substrate (Sacchi et al., 2004; Molla et al., 2006). The initial reaction rates (μmol/min) at different substrate concentrations (in the 0.1–10-fold *K*_m_ range) are fitted using the Michaelis-Menten equation:

$$\begin{array}{l} v=\frac{{\text{V}}_{\max}\;\times \;\left[\text{S}\right]}{K_{\text{m}}+\left[\text{S}\right]} \end{array}$$

where V_max_ and *K*_m_ are the apparent (i.e., at fixed O_2_ concentration, 0.253 mM) maximal velocity and Michaelis-Menten constant for the substrate tested.

### Determination of the redox dye reduction

This assay is based on the reduction of one of the redox dyes DCPIP, MG, and Thi by DAAO. These dyes show absorption changes between their oxidized (colored) and reduced (colorless) forms in the visible range, with absorption maxima at 520, 655, and 600 nm, respectively (Brugger et al., 2014).

1. The reaction mixture is prepared in a 96-well microplate, mixing the electron acceptor solution (450 μM DCPIP, 75 μM MG, or 100 μM Thi, all final concentrations) and the D-amino acid solution in 50 mM phosphate buffer, pH 8.0, in a total volume of 100 μL.
2. 10–20 μL of DAAO are added (~0.2 U).
3. The electron acceptor reduction is followed at 520 nm for DCPIP, 655 nm for MG, and 600 nm for Thi (Abs_x__nm_).
4. The DAAO activity is calculated from the initial absorbance variation at the corresponding wavelength per minute (ΔAbs_x__nm_/min) using the following equation:
  $$\begin{array}{l} \frac{\text{U}}{\text{m}{\text{L}}_{\text{DAAO}}}=\frac{{\text{ΔAbs}}_{\text{x nm}}/\text{min}}{\varepsilon_{\text{x nm }\left(\text{redox dye}\right)\;}\;\times \text{m}{\text{L}}_{\text{DAAO}}}\;\times \text{m}{\text{L}}_{\text{TOT}} \end{array}$$
  where the molar extinction coefficients (ε_x__nm_) of DCPIP, MG, and Thi are 6.6, 46.6, and 55.4 mM^−1^cm^−1^, at 520, 655, and 600 nm, respectively. mL_TOT_ is the final volume of the reaction mixture (0.1 mL). For an accurate determination of the DAAO activity, the ΔAbs_x__nm_/min should be in the 0.1–0.3 range. The detection range is reported in Table **3**.

### Determination of hydrogen peroxide production

**A) Horseradish Peroxidase and** ***o*-Dianisidine Coupled Assay**

The hydrogen peroxide produced by the DAAO reaction is reduced by HRP to water and *o*-DNS is simultaneously oxidized to give a colored compound showing an absorption maximum at 440 nm with an extinction coefficient of 13 mM^−1^cm^−1^ (see Figures 1, 2A).

1. The reaction mixture is prepared in a plastic disposable microcuvette or in a 96-well microplate, mixing the D-amino acid solution, 1 mM *o*-DNS, and 1 U/mL of HRP, in 75 mM disodium pyrophosphate buffer, pH 8.5 (all final concentrations), in a total volume of 1 mL or 300 μL, for microcuvettes or 96-well microplates, respectively.
2. 10–20 μL of DAAO are added (~0.01 U).
3. The absorbance at 440 nm (Abs_440__nm_) is monitored.
4. The DAAO activity is calculated from the initial absorbance variation at 440 nm per minute (ΔAbs_440__nm_ /min) using the following equation:
  $$\begin{array}{l} \frac{\text{U}}{\text{m}{\text{L}}_{\text{DAAO}}}=\frac{{\text{ΔAbs}}_{440\text{ nm}}/\text{min}}{\varepsilon_{440\text{ nm }\left(o-\text{DNS}\right)\;}\;\times \text{m}{\text{L}}_{\text{DAAO}}}\;\times \text{m}{\text{L}}_{\text{TOT}} \end{array}$$
  where the molar extinction coefficient at 440 nm of *o*-DNS is 13 mM^−1^cm^−1^ and mL_TOT_ is the final volume of the reaction mixture (1 or 0.3 mL). To accurately determine the DAAO activity, the Δ*Abs*_440 nm_/min should be in the 0.1–0.3 range. The detection range is reported in Table **3**.

#### Application: screening for RgDAAO variants with broader substrate specificity

**Figure 6:**
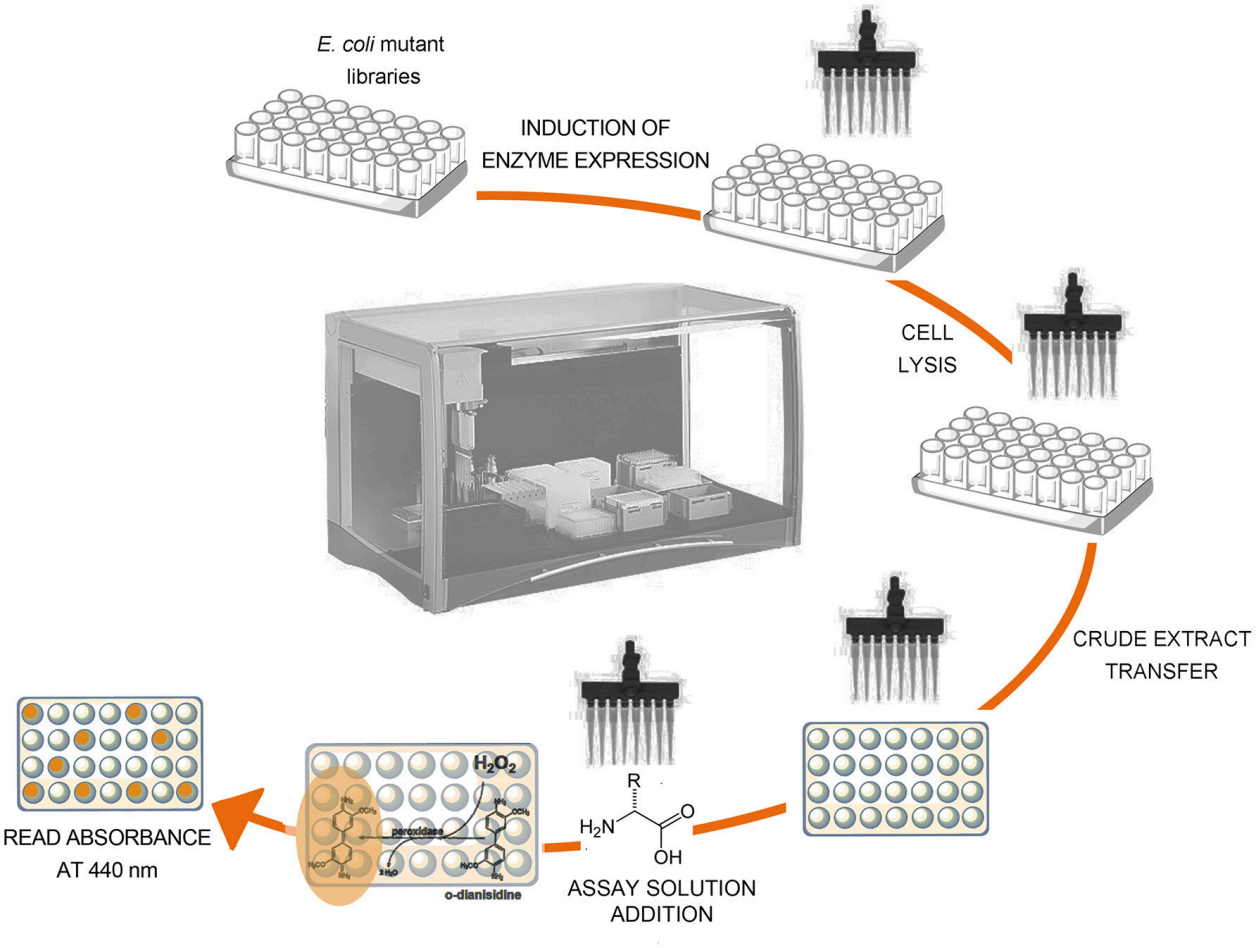
Schematic representation of the screening procedure on crude extract of *E. coli* cells expressing RgDAAO variants.

The reaction catalyzed by RgDAAO has been exploited in the analytical determination of the D-amino acid content in biological samples (Rosini et al., 2008). Since the enzyme is not active on acidic D-amino acids, it cannot be used to detect the total amount of D-amino acids. In an attempt to obtain DAAO variants to detect and quantify the total amount of D-amino acids, a directed evolution approach was performed; the wild-type DAAO cDNA was randomly mutated by error-prone PCR and a colorimetric screening procedure was set up to determine any alteration in the substrate specificity of RgDAAO (Sacchi et al., 2004). This screening procedure is performed in an automated liquid handling system (see Figure 6):

1. To the 1 mL saturated *E. coli* culture 1 mM isopropyl β-D-thiogalactopyranoside (IPTG, final concentration) is added and then incubated at 30°C for 2 h.
2. 50 μL of the cells culture are transferred to different wells of a 96-well microplate (one microplate for each substrate tested).
3. 150 μL of lysis buffer are added in each well and the plates incubated at 37°C for 30 min.
4. DAAO activity is assayed on the crude extracts by adding 100 μL of developing solution (100 mM D-amino acid, 1 mM *o*-DNS and 1 U/mL HRP, in 100 mM disodium pyrophosphate buffer, pH 8.5, all final concentrations).
5. The time course of the absorbance change at 440 nm is followed at room temperature by using a microtiter plate reader and comparing it with that of cells expressing wild-type RgDAAO as control.

**(B) Horseradish Peroxidase and 4-Aminoantipyrine Coupled Assay**

This assay determines the amount of hydrogen peroxide produced by the reaction catalyzed by DAAO coupled to the oxidation of 4-AAP by HRP in the presence of phenol. The resulting quinoneimine product shows an absorption maximum at 505 nm with an extinction coefficient of 6.58 mM^−1^cm^−1^ (see Figure 2B).

1. The reaction mixture is prepared in a plastic disposable microcuvette or in a 96-well microplate, mixing the D-amino acid solution, 1.5 mM 4-AAP, 2 mM phenol, and 2.5 U/mL HRP, in 75 mM disodium pyrophosphate buffer, pH 8.5 (all final concentrations), in a total volume of 1 mL or 300 μL, for microcuvettes or 96-well microplates, respectively.
2. 10–20 μL of DAAO are added (~0.05 U).
3. The absorbance at 505 nm (Abs_505__nm_) is monitored.
4. The DAAO activity is calculated from the initial absorbance variation at 505 nm per minute (ΔAbs_505__nm_ /min) using the following equation:
  $$\begin{array}{l} \frac{\text{U}}{\text{m}{\text{L}}_{\text{DAAO}}}=\frac{2\;\times \;{\text{ΔAbs}}_{505\text{ nm}}/\text{min}}{\varepsilon_{505\text{ nm }\left(\text{quinoneimine}\right)\;}\;\times \text{m}{\text{L}}_{\text{DAAO}}}\;\times \text{ m}{\text{L}}_{\text{TOT}} \end{array}$$
  where the molar extinction coefficient at 505 nm of quinoeimine is 6.58 mM^−1^ cm^−1^ and mL_TOT_ is the final volume of the reaction mixture (1 or 0.3 mL). To accurately determine the DAAO activity, the Δ*Abs*_505 *nm*_/min should be in the 0.1–0.3 range. The detection range is reported in Table **3**. Owing to the stoichiometry of the reaction, in which two molecules of hydrogen peroxide react with 4-AAP to produce one molecule of quinoneimine (see Figure 2B), a factor 2 is added at the numerator.

**C) Horseradish Peroxidase and Amplex**^®^ **UltraRed Coupled Assay**

This assay is based on the reaction catalyzed by HRP on the fluorogenic substrate Amplex® UltraRed with hydrogen peroxide in a 1:1 stoichiometric ratio to give the brightly fluorescent product Amplex® UltroxRed (see Figure 2C).

General procedure:

1. The reaction mixture, as described for each application below, is prepared and set up in a 96-well microplate.
2. The microplate is incubated for 30–60 min in the dark at 25°C under agitation.
3. The Stop Reagent solution is added to each well and the microplate is incubated for 5 min in the dark at 25°C under agitation. This step allows the fluorescence signal-generating reaction to be terminated at a user-determined time point, ensuring that the timing of the standard and unknown samples measurements is the same. After reagent addition, the fluorescence signal is stable for at least 3 h.
4. The fluorescence intensity is recorded using a fluorimeter microplate reader: λ_exc_ = 535 nm (slit = 25 nm); λ_em_ = 595 nm (slit = 20 nm).
5. The enzymatic activity is calculated on the basis of a calibration curve obtained using known amounts of purified recombinant DAAO (0.02–0.4 mU range). The detection range is reported in Table **3**. Due to the response variability of the fluorescence signal, the calibration curve must be carried out on each 96-well microplate, and triplicates for each standard and sample are recommended.

#### Applications

***(a) Detection of DAAO Activity on Different D-Amino Acids of Purified Protein*** ***Preparations***

1. Preparation of the reaction mixture: 50 μL of WS_A_ (0.1 mM Amplex® UltraRed Reagent, 0.5 U/mL HRP, in 50 mM sodium phosphate buffer, pH 7.4), 45 μL of D-amino acid solution, and 5 μL of DAAO (~1 mU) are mixed in each well;
2. Reaction is stopped by adding 20 μL of Stop Reagent solution after 30 min.

***(b) Detection of DAAO Activity in U87 Cells***

The enzymatic assay is performed on U87 glioblastoma control (not transfected) cells and on the same cells transfected with the plasmid expressing hDAAO (Sacchi et al., 2011).

1. The U87 cells are resuspended in lysis buffer (4 × 10^5^ cells per mL) and disrupted by sonication.
2. The soluble fraction is collected by centrifugation at 16,000 × *g* for 30 min at 4°C.
3. Preparation of the reaction mixture: 50 μL of cell extract (the supernatant obtained in the previous step) and 50 μL of WS_B1_ (40 mM D-serine, 50 μM FAD, 10 mM sodium azide, 0.1 mM Amplex® UltraRed Reagent, 0.2 U/mL HRP, in 50 mM sodium phosphate buffer, pH 7.4) are mixed in each well.
  On each microplate, three negative controls must be set up:
  – *control I* (without cell extract): 50 μL of lysis buffer and 50 μL of WS_B1_ are mixed in each well;
  – *control II* (without substrate): 50 μL of cell extract and 50 μL of WS_B2_ are mixed in each well;
  – *control III* (with a DAAO inhibitor): 50 μL of cell extract and 50 μL of WS_B3_ are mixed in each well.
4. Reaction is stopped by adding 20 μL of Stop Reagent solution after 60 min.
5. The fluorescence intensity values should be similar for all three controls (background value) and should be 2–3% of the values of the samples. The fluorescence values of the samples (with both cell extract and substrate) are subtracted from those recorded for *control II* (with cell extract and without substrate).

***(c) Identification of hDAAO Inhibitors***

The effect of small molecules on the enzymatic activity of hDAAO is evaluated by measuring the activity of purified recombinant hDAAO at different concentrations of inhibitors, in the presence of D-serine as substrate (Hopkins et al., 2013; Terry-Lorenzo et al., 2014, 2015).

1. The preincubation mixtures are prepared in Eppendorf-type test tubes mixing 25 μL of 0.01 mg/mL hDAAO with 625 μL of different inhibitors (in the 0–1.25 mM final concentration range), in 50 mM sodium phosphate buffer, pH 7.4, 4 μM FAD.
2. The tubes are incubated for 30 min at room temperature.
3. Preparation of the reaction mixture: 130 μL of preincubation mixture are transferred to each well of a microplate (four wells for each inhibitor's concentration tested) and 70 μL of WS_C_ (22 mM D-serine, 4 μM FAD, 0.1 mM Amplex® UltraRed Reagent, 0.2 U/mL HRP, in 50 mM sodium phosphate buffer, pH 7.4) are added.
4. Reaction is stopped by adding 40 μL of Stop Reagent solution after 30 min.
5. The fluorescence value determined for the control in the absence of the compound is used as reference (activity = 100%).
6. Data are fit to a standard, four-parameter equation to determine curve top, bottom, inhibitor concentration giving 50% inhibition (IC_50_), and Hill Slope:
  $$\begin{array}{l} y=\text{Bottom}+\frac{\left(\text{Top}-\text{Bottom}\right)}{{\left(1+10\;ˆ\left(\left(\text{LogI}{\text{C}}_{50}-x\right)\times \text{Hill slope}\right)\right)}_{\;}} \end{array}$$
  where Bottom is the fluorescence value at the bottom plateau and Top is the fluorescence value at the top plateau (Terry-Lorenzo et al., 2015).

**(D) Catalase and Purpald**^®^ **Coupled Assay**

The hydrogen peroxide produced by the DAAO reaction is reduced to water by catalase in the presence of methanol as hydrogen donor, giving formaldehyde that, in turn, reacts with the chromogenic compound Purpald® to give the colored product 6-mercapto-*s*-triazolo-[4,3-*b*]-*s*-tetrazine showing an absorption maximum at 550 nm with an extinction coefficient of 7.74 mM^−1^cm^−1^ (Dickinson and Jacobsen, 1970; Watanabe et al., 1978; Sasabe et al., 2014; see Figure 3).

1. The reaction mixture is prepared in a plastic tube mixing 300 μL of the D-amino acid solution, 200 μL of 0.1 mM FAD, 300 μL of 700 U/mL catalase, in 133 mM sodium pyrophosphate buffer, pH 8.3, and 100 μL of 70% methanol (final volume: 900 μL).
2. 100 μL of DAAO solution are added (~0.02 U).
3. The reaction mixture is incubated at 37°C for 15 min under agitation.
4. The reaction is stopped by adding 1 mL of 10% (w/v) trichloroacetic acid (final volume: 2 mL) followed by centrifugation at 700 × *g* for 20 min.
5. A blank solution is prepared, adding 1 mL of 10% (w/v) trichloroacetic acid before the DAAO solution is added.
6. To 1 mL of the supernatant from step 4, 1 mL of 5 M KOH and 1 mL of 34 mM Purpald® in 0.5 M HCl are added.
7. The solution from step 6 is incubated at room temperature for 15 min, followed by the addition of 1 mL of 0.75% (w/v) KIO_4_ in 0.2 M KOH with vigorous shaking.
8. 1 mL of the solution from step 7 is transferred in a plastic disposable microcuvette and the absorbance at 550 nm (Abs_550__nm_) is measured.
9. The absorbance at 550 nm is corrected by subtracting the absorbance value of a blank solution and the DAAO activity is calculated from the ΔAbs_550nm_ using the following equation (Watanabe et al., 1978):
  $$\begin{array}{l} \frac{\text{U}}{\text{m}{\text{L}}_{\text{DAAO}}}=\frac{2\;\times \;{\text{ΔAbs}}_{550\text{ nm}}/15\text{ min}}{\varepsilon_{550\text{ nm }\left(\text{chromogen}\right)\;}\;\times \text{m}{\text{L}}_{\text{DAAO}}}\;\times \text{ m}{\text{L}}_{\text{TOT}} \end{array}$$
  where the molar extinction coefficient at 550 nm of 6-mercapto-*s*-triazolo-[4,3-*b*]-*s*-tetrazine is 7.74 mM^−1^cm^−1^ and mL_TOT_ is the final volume of the solution from step 7 (4 mL). The detection range is reported in Table **3**. In this assay an average velocity corresponding to the first 15 min of the reaction is determined. A factor 2 is added at the numerator because only half of the initial sample (from step 4) is reacted with Purpald®.

#### Application: detection of DAAO activity in tissues

The enzymatic assay is performed on post mortem human brain tissues dissected after perfusion from the thoracic aorta with ice-cold phosphate buffer saline (PBS), pH 7.4 (Sasabe et al., 2014).

1. The tissue is homogenized in 7 mM sodium pyrophosphate buffer, pH 8.3, at 3,500 rpm for 2 min and centrifuged at 5,500 × *g* for 10 min at 4°C. The supernatant (tissue lysate) is recovered and the pellet discarded.
2. 50 μL tissue lysate is added to the reaction mixture containing 150 μL of 100 mM D-alanine, 100 μL 0.1 mM FAD, 150 μL of 700 U/mL catalase in 133 mM sodium pyrophosphate buffer, pH 8.3, and 50 μL of 70% (v/v) methanol.
3. The reaction mixture is incubated at 37°C for 60 min under stirring.
4. Reaction is stopped by adding 500 μL of 10% (w/v) trichloroacetic acid and the reaction mixture is centrifuged at 700 × *g* for 20 min. The supernatant is recovered and the pellet discarded.
5. To 250 μL of the supernatant solution 250 μL of 5 M KOH and 250 μL of 34 mM Purpald® in 0.5 M HCl are added.
6. The reaction mixture is incubated at room temperatures for 15 min to which 250 μL 0.75% (w/v) KIO_4_ in 0.2 M KOH is added under vigorous shaking.
7. The DAAO activity is measured on the supernatant and expressed as the amount of D-alanine oxidized per min per milligram of total proteins. Total protein concentration is determined by the method of (Lowry et al., 1951).

### Determination of α-Keto acid production

**A) Direct Spectrophotometric Assay**

1. Preparation of reaction mixture: 990 μL of D-amino acid solution in 75 mM disodium pyrophosphate buffer, pH 8.5, are transferred into a quartz microcuvette.
2. The initial absorbance value at the wavelength corresponding to the maximum absorption peak of the α-keto acid produced from the starting amino acid is recorded.
3. Measurement of DAAO activity: 10 μL of DAAO (~0.01 U) are added and the increase of absorbance at the selected wavelength, due to α-keto acid production, is recorded. The DAAO activity is calculated from the ΔAbs/min using the following equation:
  $$\begin{array}{l} \frac{\text{U}}{\text{m}{\text{L}}_{\text{DAAO}}}=\frac{{\text{ΔAbs}}_{\text{x nm}}/\text{min}}{\varepsilon_{\text{x nm }\left(\text{α}-\text{keto acid}\right)\;}\;\times \text{m}{\text{L}}_{\text{DAAO}}}\;\times \text{ m}{\text{L}}_{\text{TOT}} \end{array}$$
  where ε_x__nm_ is the extinction coefficient at the selected wavelength of the α-keto acid produced and mL_TOT_ is the final volume of the reaction mixture (1 mL). The detection range is reported in Table **3**.
4. When the extinction coefficient of the α-keto acid is unknown, a calibration curve is set up by recording the absorbance values at known concentrations of the α-keto acid.

**(B) Indirect Assay with 2,4-Dinitrophenylhydrazine**

1. Preparation of DAAO reaction mixture: 10 μL of DAAO (~0.05 U) is added to the D-amino acid solution in 75 mM disodium pyrophosphate buffer, pH 8.5 (final volume = 0.3 mL), and the reaction mixture is incubated at 25°C for 10 min.
2. Derivatization of α-keto acid produced by DAAO reaction with DNP (Figure 4): 0.15 mL of 1 mM DNP, solubilized in 1 M HCl, are added to the reaction mixture.
3. The reaction is stopped by adding 1.05 mL of 0.6 M NaOH, followed by incubation at 25°C for 5 min to allow color development.
4. A blank solution is prepared using the same procedure, but without substrate (D-amino acid).
5. The absorbance at 445 nm of the reaction mixture is corrected by subtracting the absorbance value of blank solution and the DAAO activity is calculated from the ΔAbs_445 nm_ using the following equation:
  $$\begin{array}{l} \frac{\text{U}}{\text{m}{\text{L}}_{\text{DAAO}}}=\frac{{\text{ΔAbs}}_{445\text{ nm}}/10\text{ min}}{\varepsilon_{\#x000A0;445\text{ nm }\left(\alpha -\text{keto acid derivative}\right)\;}\;\times \text{m}{\text{L}}_{\text{DAAO}}}\times \text{m}{\text{L}}_{\text{TOT}} \end{array}$$
  where ε_445__nm_ is the molar extinction coefficient at 445 nm of the α-keto acid derivative and mL_TOT_ is the final volume of the reaction mixture. In this assay an average velocity corresponding to the first 10 min of the reaction is determined. The detection range is reported in Table **3**. If the extinction coefficient of the 2,4-dinitrophenylhydrazone derivative of the α-keto acid is unknown, a calibration curve is set up by recording the absorbance values obtained upon derivatization of different known concentrations of the α-keto acid with DNP under the same experimental conditions.

### Determination of ammonium production: glutamate dehydrogenase and α-ketoglutarate coupled assay

1. Preparation of the reaction mixture: D-amino acid solution, 5 mM α-ketoglutarate, 0.25 mM NADH, and 10 U/mL of GDH, in 75 mM disodium pyrophosphate buffer, pH 8.5 (all final concentrations).
2. 1 mL of this solution is transferred to a quartz microcuvette and the absorbance at 340 nm of the reaction mixture is recorded.
3. Measurement of DAAO activity: 10 μL of DAAO (~1 U) are added and the decrease in absorbance at 340 nm, due to the conversion of NADH to NAD^+^ by GDH reaction, is proportional to the amount of ammonium produced by DAAO reaction. The DAAO activity is calculated from the ΔAbs_340__nm_/min using the following equation:
  $$\begin{array}{l} \frac{\text{U}}{\text{m}{\text{L}}_{\text{DAAO}}}=\frac{{\text{ΔAbs}}_{340\text{ nm}}/\text{min}}{\varepsilon_{340\text{ nm }\left(\text{NADH}\right)\;}\;\times \text{m}{\text{L}}_{\text{DAAO}}}\;\times \text{ m}{\text{L}}_{\text{TOT}} \end{array}$$
  where the molar extinction coefficient at 340 nm of NADH is 6.3 mM^−1^cm^−1^ and mL_TOT_ is the final volume of the reaction mixture. The detection range is reported in Table **3**.

## Anticipated results

### Determination of the steady-state kinetic parameters and substrate specificity

**Figure 7:**
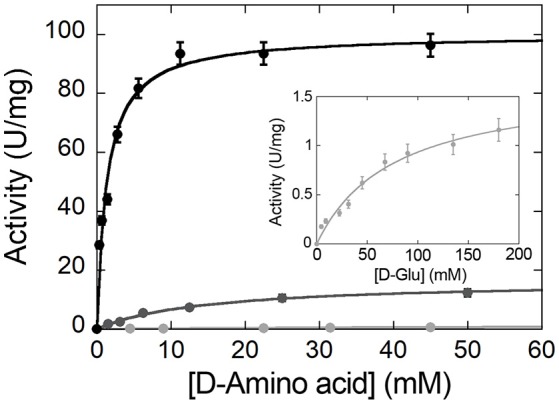
Michaelis-Menten plot of the kinetics of wild-type RgDAAO on D-alanine (black), D-lysine (gray), and D-glutamate (light gray and inset) as substrate, at pH 8.5 and 25°C. All measurements were performed at air saturation ([O_2_] = 0.253 mM) using the oxygen consumption assay.

The apparent (i.e., at fixed O_2_ concentration) kinetic parameters (*k*_cat__, app_ and *K*_m__, app_ values) at air saturation ([O_2_] = 0.253 mM at 25°C) are determined for the wild-type RgDAAO enzyme on various D-amino acids using the oxygen consumption assay (Sacchi et al., 2004). As shown in Figure 7, the chemical nature of the substrates profoundly influences the kinetic parameters; a 6- and 60-fold lower activity is apparent on basic and acidic substrates (D-lysine and D-glutamate, respectively) in comparison to the value obtained on the neutral D-alanine (see Table 2).

**Table 2:** Comparison of the apparent kinetic parameters of RgDAAO on different substrates.

	**V_max_ (U/mg)**	***K*_m_ (mM)**	**V_max_/*K*_m_**
D-Alanine	100 ± 3	1.3 ± 0.2	76.9
D-Lysine	16.4 ± 0.5	14.6 ± 1.4	1.1
D-Glutamate	1.7 ± 0.1	76.4 ± 6.4	0.02

### Screening for RgDAAO variants with broader substrate specificity

**Figure 8:**
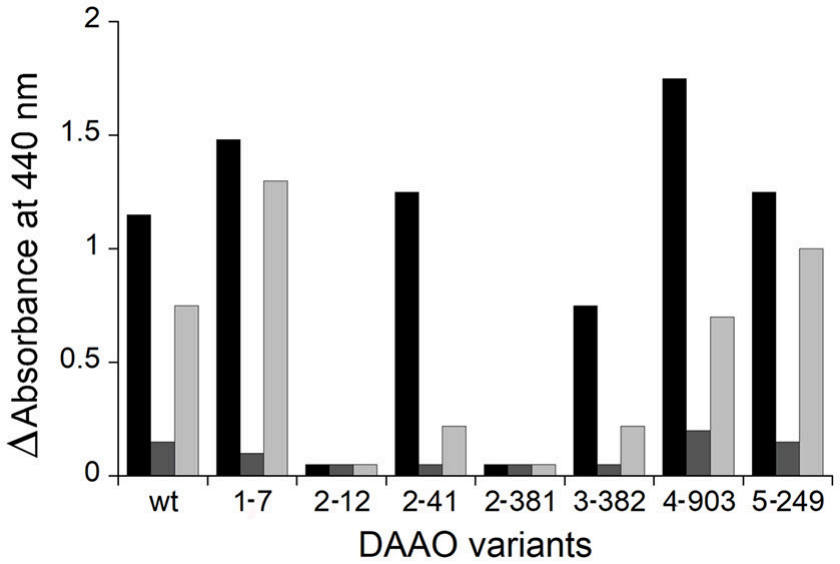
Comparison of DAAO activity determined by the coupled reaction with HRP and *o*-DNS and expressed as absorbance change at 440 nm of wild-type and variants RgDAAO identified following five rounds of random mutagenesis and determined on D-alanine (black), D-aspartate (gray), and D-arginine (light gray) as substrates (30 mM final concentration). Figure obtained on the basis of the results published in Sacchi et al. (2004).

In an attempt to evolve the substrate specificity of RgDAAO, five rounds of random mutagenesis (under different PCR reaction conditions) are performed (Sacchi et al., 2004): a rapid and sensitive assay is required to select the clone expressing the variant with the activity of interest. By using the colorimetric assay based on the reaction of RgDAAO on the D-amino acids of interest coupled to HRP and *o*-DNS, RgDAAO variants showing an altered substrate specificity could be identified (Sacchi et al., 2004). The crude extract of *E. coli* culture expressing clones 1-7 and 2-41, deriving from the first and the second set of PCR conditions, respectively, showed a similar activity on D-alanine, but an altered activity on D-arginine with respect to the wild-type enzyme (Figure 8). Furthermore, clones 4-903 and 5-249, deriving from the fourth and the fifth set of PCR conditions, respectively, expressed RgDAAO variants showing a higher activity on at least two of the three substrates tested (D-alanine, D-aspartate, and D-arginine) compared to the wild-type enzyme (see Figure 8). These four RgDAAO variants possess a broader substrate specificity and thus are useful for analytically determining the total content of D-amino acids.

### Identification of hDAAO inhibitors

**Figure 9:**
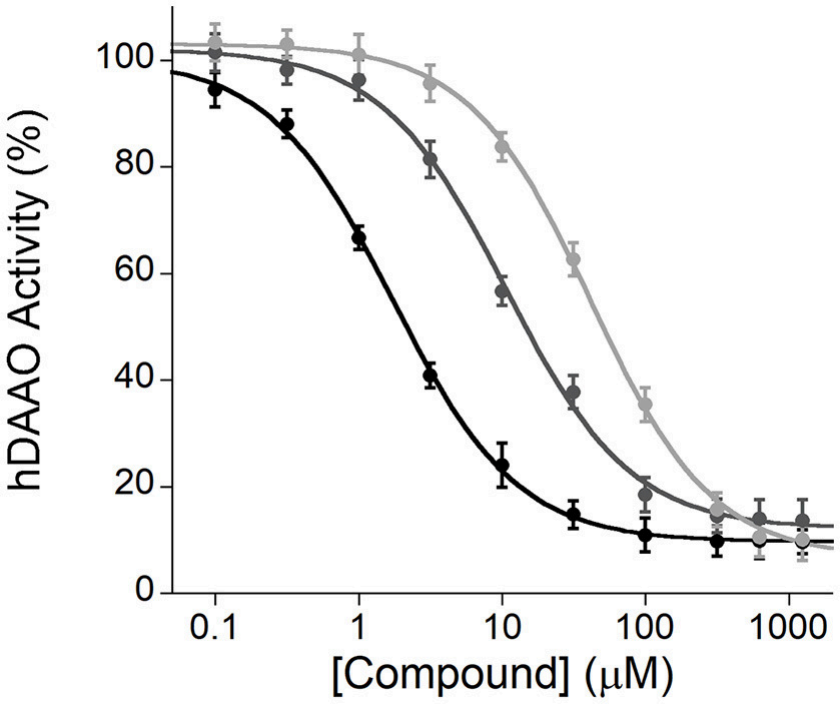
Enzyme inhibition assays performed by the Amplex® UltraRed method using recombinant hDAAO, D-serine as substrate and three different compounds (1, black; 2, gray; and 3, light gray) as potential inhibitors. The plot displays the mean values ± SD, *n* = 4.

The Amplex® UltraRed-based fluorescence enzyme assay is a useful and adequate screening technique to identify novel inhibitors of hDAAO. As stated in the Introduction section, this flavoenzyme controls the levels of D-serine in the brain, the coagonist of the NMDARs, a central regulator of synaptic plasticity, learning, and memory (Panatier et al., 2006; Zhou and Sheng, 2013): the inhibitors of hDAAO have the potential to treat nervous system disorders such as schizophrenia (Sacchi et al., 2013). Computational tools are used to identify libraries of compounds, then screened for hDAAO inhibition. The affinity of the different molecules is based on IC_50_ values (i.e., the compound concentration at which hDAAO activity is halved). As shown in Figure 9, all compounds tested inhibited the enzymatic activity, although compound 1 was a more potent inhibitor compared to compounds 2 and 3 (IC_50_ = 1.7 ± 0.1, 10.6 ± 0.9 and 43.5 ± 2.3 μM, respectively).

### Detection of DAAO activity in tissues

**Figure 10:**
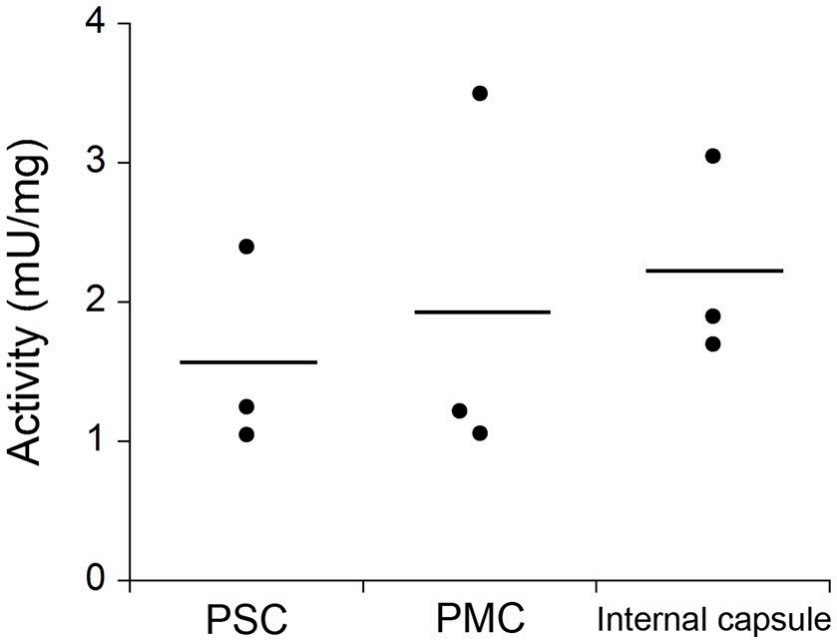
Comparison of DAAO activity in different human CNS samples determined by catalase and Purpald® coupled assay: primary somatosensory cortex (PSC), primary motor cortex (PMC), and the posterior limb of internal capsule. DAAO activity is expressed as milliunits per milligram of total proteins (mU/mg). Statistical analyses were performed with ANOVA, *n* = 3. Figure obtained on the basis of the results published in Sasabe et al. (2014).

Owing to the involvement of hDAAO in neuropsychiatric disorders (see Introduction), the investigation of its distribution based on its activity in the human CNS is of utmost importance. Thus, DAAO activity in post mortem samples from different regions of the CNS of mice and humans was determined using a coupled assay with catalase and Purpald® (Sasabe et al., 2014). DAAO activity was detected in all the human specimens examined; significant activity was found along the corticospinal tract, rubrospinal tract, nigrostriatal sytem, ponto-/olivo-cerebellar fibers, and in the anterolateral system. DAAO activity was also detected in human primary somatosensory cortex (PSC), in primary motor cortex (PMC), and in the internal capsule, whereas in mice there was no such activity in the cerebral cortex and in the striatum (Figure 10).

## Conclusions

It is useful to determine DAAO activity for different biotechnological and medical applications (Pollegioni et al., 2007b, 2008; Pollegioni and Molla, 2011): (i) investigation of kinetic parameters on different substrates in order to identify the optimized enzyme for a specific industrial process (i.e., synthesis of semi-synthetic antibiotics, resolution of racemic mixtures, etc.); (ii) identification of novel inhibitors of the human DAAO to be used as potential drugs for the treatment of neuropsychiatric disorders; (iii) evolution of DAAO variants more active at low oxygen concentration for the application in anticancer enzyme therapy; and (iv) development of enzyme-based biosensors for the analytical determination of D-amino acids content in biological fluids.

DAAO activity can be determined using direct or coupled assays which differ in terms of sensitivity, required instrumentation and cost, as described in this paper and summarized in Table 3.

**Table 3:** Comparison of different assays of DAAO activity.

**Assay**	**Type of assay**	**Type of detection**	**Sensitivity range (mU**_DAAO_**/mL)**	**Cost per assay**
Molecular oxygen consumption	Direct, continuous	Amperometry	10–100	$
Redox dye reduction	Direct, continuous	Abs at different wavelengths	500–5000	$
*o*-DNS	Coupled (HRP), continuous	Abs at 440 nm	5–25	$$
4-AAP	Coupled (HRP), continuous	Abs at 505 nm	25–100	$$
Amplex® UltraRed	Coupled (HRP), discontinuous	Fluorescence (λ_exc_ at 535 nm, λ_em_ at 590 nm)	0.25–2.5	$$$
Purpald®	Coupled (catalase), discontinuous	Abs at 550 nm	100–500	$$
α-Keto acid	Direct, continuous	Abs at λ_max_	5–25	$
DNP	Chemical derivatization, discontinuous	Abs at 445 nm	100–500	$
Ammonium production	Coupled (GDH), continuous	Abs at 340 nm	500–2,500	$$

## Notes

– Assay buffers must be pre-equilibrated at the working temperature.
– Instruments with similar specifications of the ones listed in “Materials and Equipment” section can be used.
– The reaction rate can be increased by incubating the reaction mixtures at higher temperature (e.g., 37°C instead of 25°C), taking in account the thermal stability of the enzymes in the experimental conditions.
– In absorbance and fluorescence assays, a sample containing buffer instead of the substrate and another one containing buffer instead of the enzyme must be performed as negative controls to exclude the presence of artifacts. The presence of contaminants in the sample might affect the absorbance or fluorescence signals (e.g., the presence of nucleic acids and/or proteins can increase the absorbance intensity in the UV region).
– In coupled assays with HRP or GDH the production of the expected α-keto acid is not directly monitored but it can be verified by different analytical methods (*e.g*. HPLC, NMR, and MS).
– As a general rule, continuous assays should be preferred (if possible), since the initial rate estimation is easier, due to the fact that the shape of the progress curve is visible all along the assay time and any discrepancy from the linearity is thus promptly detected. On the contrary, in discontinuous assays, to assess the linearity of the initial rate, withdrawal of different samples at fixed incubation times is necessary. Furthermore, the termination of the reaction and withdrawal of samples at fixed-time can introduce time and volume inaccuracy.

## Author contributions

All authors listed have made a substantial, direct and intellectual contribution to the work, and approved it for publication.

### Conflict of interest statement

The authors declare that the research was conducted in the absence of any commercial or financial relationships that could be construed as a potential conflict of interest.

## Abbreviations

4-AAP: 4-aminoantipyrine
CNS: central nervous system
DAAO: D-amino acid oxidase
DCPIP: 2,6-Dichlorophenol-indophenol
DNaseI: desossiribonuclease I
DNP: 2,4-dinitrophenylhydrazine
EDTA: ethylenediaminetetraacetic acid
FAD/FADH_2_: flavin adenine dinucleotide oxidized/reduced form
GDH: glutamate dehydrogenase
hDAAO: human D-amino acid oxidase
HRP: horseradish peroxidase
MG: methylene green
NAD^+^/NADH: nicotinamide adenine dinucleotide oxidized/reduced form
NMDARs: N-methyl-D-aspartate receptors
*o*-DNS: *o*-dianisidine
RgDAAO: D-amino acid oxidase from *Rhodotorula gracilis*
Thi: thionine.
